# Progression to Severe Hypernatremia in Hospitalized General Medicine Inpatients: An Observational Study of Hospital-Acquired Hypernatremia

**DOI:** 10.3390/medicina56070358

**Published:** 2020-07-17

**Authors:** Ramessh Ranjan, Stacey C.-Y. Lo, Stephanie Ly, Visakan Krishnananthan, Andy K.H. Lim

**Affiliations:** 1 Monash Health General Medicine, Dandenong Hospital, 135 David Street, Dandenong, VIC 3175, Australia; ramessh.ranjan@gmail.com (R.R.); staceylocy@gmail.com (S.C.-Y.L.); steph.ly0013@gmail.com (S.L.); vishan337@hotmail.com (V.K.); 2 Department of Medicine, School of Clinical Sciences, Monash University, 246 Clayton Road, Clayton, VIC 3168, Australia

**Keywords:** hypernatremia, sodium, water imbalance, inpatients, hospital medicine, fluid therapy

## Abstract

*Background and objectives:* Hypernatremia can be community or hospital-acquired, and there may be specific factors unique to the hospital environment, such as intravenous fluid treatment, which contribute to hypernatremia. The aim of this study was to determine the factors associated with the progression from moderate to severe hospital-acquired hypernatremia among patients admitted under general medicine. *Materials and Methods:* In this retrospective, single-center cohort study (2012 to 2017), we used ICD-10 coding and medical records to identify adult patients who developed moderate hypernatremia and followed them for progression to severe hypernatremia. We profiled the serum biochemistry and the volume and composition of prescribed intravenous fluids. We applied logistic regression to determine the factors associated with the progression to severe hypernatremia, using the patients with moderate hypernatremia as reference. *Results:* Of the 180 medical inpatients (median age of 81 years) with moderate hospital-acquired hypernatremia, 9.4% progressed to severe hypernatremia. Normal saline comprised 76% of intravenous fluid volume administered prior to onset of moderate hypernatremia. After the onset, 38% of fluid volume prescribed remained normal saline. The factors independently associated with progression to severe hypernatremia included chronic kidney disease stage (odds ratio 2.38, 95% CI: 1.26–4.50, *P* = 0.008) and serum creatinine increase (per 10 µmol/L, OR 1.29, 95% CI: 1.07–1.57, *P* = 0.009). *Conclusions:* Patients with chronic kidney disease and acute kidney injury may have an increased risk of severe hospital-acquired hypernatremia.

## 1. Introduction

Sodium and water disorders are the most common electrolyte abnormalities in hospitalized patients [1]. Hypernatremia develops from either free water loss or gain of sodium, or a combination. In general, elderly and debilitated patients are prone to hypernatremia due to impaired sense of thirst and access to water, combined with a reduced concentrating ability of the kidney with aging [1]. Severe hypernatremia is associated with reduced cardiac function, insulin resistance, impaired hepatic lactate clearance, neuromuscular impairment, and cognitive dysfunction [2,3]. Hypernatremia is an independent predictor of mortality, proportional with its severity, and can be as high as 40 to 60% [4,5].

Hypernatremia in hospitalized patients may either be community-acquired as part of the admission diagnosis, or hospital-acquired, which develops during inpatient stay. Two large studies in unselected cohorts of hospitalized adults suggested that hospital-acquired hypernatremia is more common than community-acquired hypernatremia, and hypernatremia is independently associated with an increased in-hospital mortality and length of stay [6,7].

The prevalence of hypernatremia at admission is 10 times higher in patients admitted from nursing homes than from community residences [8]. Community-acquired hypernatremia was also associated with Alzheimer’s, dementia, and impaired oral intake or access to free water, and use of renin-angiotensin inhibitors [9]. The driving force behind many cases of community-acquired hypernatremia is the altered capacity for water regulation in elderly patients and the hypernatremia is usually chronic in nature. In contrast, hospital-acquired hypernatremia is acute by definition, and cannot be purely explained by the inability to self-regulate water intake and thirst response. A hospital medical ward is a controlled environment, and therefore, the development of severe hypernatremia after admission could be viewed as an iatrogenic matter, either due to poor recognition or inadequate treatment [10,11].

While there are good studies examining the causes of community-acquired hypernatremia, there are fewer studies which have examined hospital-acquired hypernatremia, or such studies have not made a distinction between the two. We believe that the processes associated with the development of hospital-acquired hypernatremia are different to community-acquired cases, and they should be analyzed separately.

Traditionally, the peak serum sodium is considered the most important risk factor for mortality [6,12,13]. The consequences of mild hypernatremia are fairly limited if recognized and treated appropriately. The typical general medicine patient is also elderly, where few or mild symptoms develop until the serum sodium reaches 160 mmol/L [1,14]. It is unclear to us why some patients develop severe hypernatremia in hospital, while others stabilize or improve. Thus, in this study of hospitalized patients, we thought it would be relevant to specifically compare patients who developed severe hypernatremia (peak serum sodium 160 mmol/L or greater) with those who only developed moderate hypernatremia (peak serum sodium 150 to 159 mmol/L) during hospitalization. In this context, the nature of the intravenous fluid prescription would be highly relevant.

The aim of this study was to determine if intravenous fluid prescriptions and other potential risk factors were associated with the progression to severe hypernatremia in patients who had already developed moderate hypernatremia during hospitalization.

## 2. Materials and Methods

### 2.1. Study Design, Setting, and Patients

This study was based at Dandenong Hospital in the state of Victoria, Australia. Dandenong Hospital is a 573-bed acute hospital within the Monash Health network, the largest hospital network in the state, located in the southeast region of Melbourne. The General Medicine unit consists of a 24-bed Acute Assessment Unit and four ward-based units, managing up to 24 patients each. An additional medical unit is active as a winter strategy to manage additional patients. We used a cohort study design, for admissions between May 2012 to May 2017. We used the ICD-10-AM discharge diagnosis coding to identify patients with hypernatremia admitted under general medicine. We did not include surgical or specialty medicine patients to avoid heterogeneity of the study population. Specialty medicine patients often have single organ problems and a different risk profile for hypernatremia. The exclusion criteria were patients under 18 years old, patients with an initial admission serum sodium ≤125 or ≥145 mmol/L, and patients with inadequate biochemistry results for profiling. This study was approved by the Monash Health Human Research Ethics Committee as a quality initiative (RES-18-0000-671Q).

### 2.2. Study Variables and Definitions

Hypernatremia was defined based on the peak serum sodium: mild (146 to 149 mmol/L), moderate (150 to 159 mmol/L), and severe (160 mmol/L and over). The biochemistry profile collected included serum sodium and creatinine at (1) admission; (2) onset of moderate hypernatremia; (3) peak hypernatremia; and (4) discharge. Basic demographic data collected included: age, sex, residential status, functional disability (ambulant vs. non-ambulant), and included relevant comorbidities of dementia, diabetes mellitus, and chronic kidney disease (CKD) based on the CKD-EPI estimated glomerular filtration rate (eGFR).

The onset of moderate hypernatremia (sodium 150 mmol/L) was used as a reference point for baseline data collection. Data collected over seven days prior to the sodium reaching 150 mmol/L included: intensive care (ICU)—encounter (ICU stay of >24 h), duration of delirium (days), sepsis per Sepsis 3 definition [14,15], duration of fasting, diuretic use, gastrointestinal loss (vomiting or diarrhea), prescription of an oral thickened fluid regimen (based on Australian standardized definitions: level 150, 400 or 900) [16], enteral or parenteral feeding, and intravenous fluid prescription.

We then followed all patients after the onset of moderate hypernatremia for the development of severe hypernatremia (progression as the main outcome, such patients are “progressors”) and profiled the serum biochemistry and intravenous fluid prescription. Intravenous fluid data included the quantity of fluids given as their absolute and relative amounts. Intravenous fluids were regarded in dichotomous terms as normal saline (0.9% sodium chloride) or low saline, comprising all other fluid compositions with low salt solutions, inclusive of saline-free solutions such as 5% glucose. Diuretic use was examined in terms of duration and route (intravenous or oral furosemide).

### 2.3. Statistical Analysis

We used logistic regression to determine the factors associated with the development of severe hypernatremia (progressors), using patients who did not progress to severe hypernatremia (non-progressors) as the reference group. The final multivariable logistic regression model was constructed using a backwards elimination method by initially including all variables with a *P* < 0.20 in the univariable analysis. A test for interaction was considered significant at the 1% level. We compared models using Information Criteria (Akaike’s and Bayesian). We examined the model residuals to detect outliers and Pregibbon’s influence statistic to detect influential observations. We determined the model calibration (Hosmer–Lemeshow) and discrimination (c-statistic) prior to post-estimation predictions. All analysis was performed using STATA version 15.1 (StataCorp, College Station, TX, USA). A *P* < 0.05 was considered statistically significant.

## 3. Results

### 3.1. Patient Characteristics

**Figure 1:**
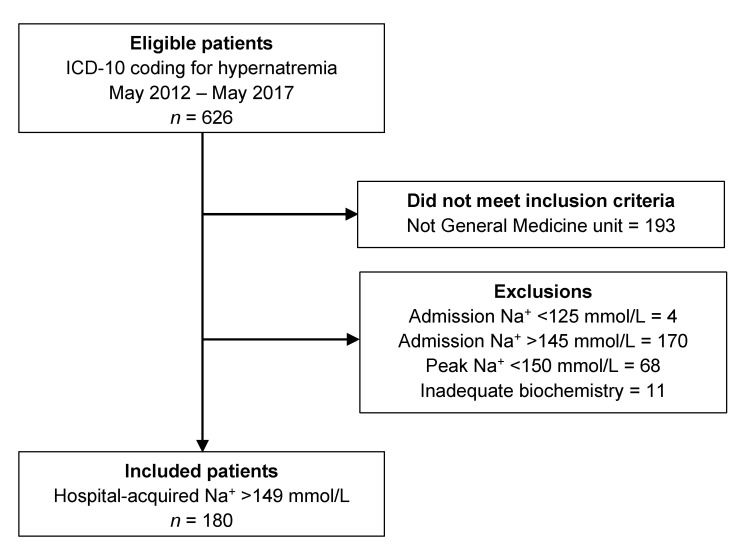
Study flow diagram showing the results of the ICD-10 search, the number meeting inclusion criteria, and the detailed reasons for exclusion.

A study flow diagram is shown in Figure 1. A total of 180 patients were included in the final analysis. The characteristics of the included patients, broken down by progression of hypernatremia status (non-progressors vs. progressors), are summarized in Table 1. The majority of patients were elderly and there was a slight male predominance. Approximately one-third were living in residential care. The median hospital length of stay was 16 days (interquartile range [IQR], 10–27 days), with a mean length of stay of 20.2 days (standard deviation [SD], 14.5 days).

There were some differences in the characteristics of progressors compared with non-progressors. Patients who progressed to severe hypernatremia were marginally older and had higher proportions with Stage 4 or 5 CKD (eGFR < 30 mL/min/1.73 m^2^). There were no obvious differences in distribution between the two groups in regard to patients who had diabetes or a functional disability. Although overall diuretic use was not different, there was a slightly higher proportion of intravenous furosemide used (compared to oral diuretics) in progressors compared with non-progressors. The isolated use (without loop diuretics) of low dose thiazide diuretics (4/180) or other diuretics acting on the distal tubules (5/180) was uncommon and could not be specifically analyzed.

### 3.2. Trends in Serum Sodium and Creatinine

**Figure 2:**
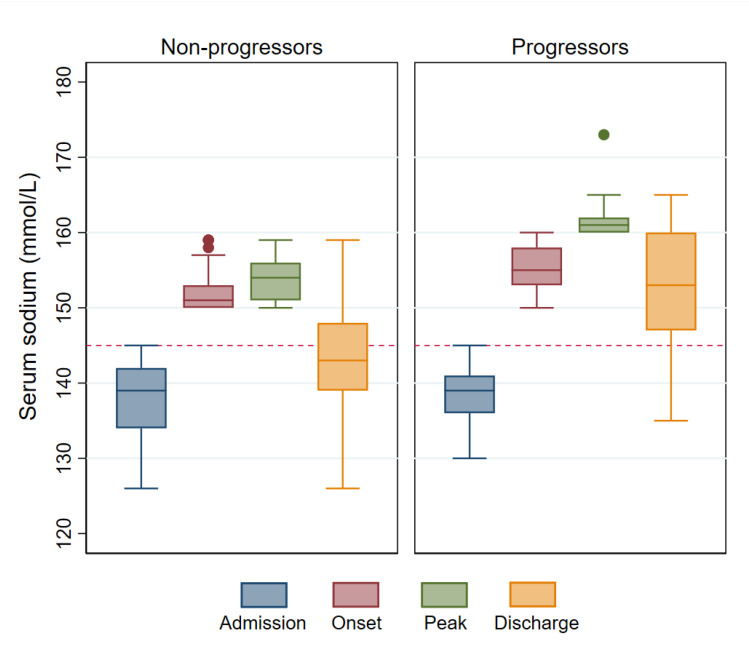
Comparison of serum sodium trends in progressors vs. non-progressors. Serum sodium stabilized in most patients after reaching 150 mmol/L (left panel) but progression to severe hypernatremia occurred in a subgroup (right panel). The horizontal dashed red line represents the upper limit of a normal serum sodium concentration (145 mmol/L).

Initially, serum sodium reached 150 mmol/L after a mean of 7.3 days (SD, 6.7 days) from admission. Subsequently, 9.4% (17/180) of patients progressed to develop severe hypernatremia. There were differences in the biochemical profile between progressors and non-progressors (Table 2). Admission serum sodium was similar in the two groups, but the serum creatinine was higher, on average, in the progressors. In non-progressors, the peak serum sodium was close to 150 mmol/L, indicating that serum sodium did not rise further in this subgroup. On the other hand, among progressors, the serum sodium continued to increase after the onset of moderate hypernatremia and peaked five days later, on average. Furthermore, serum sodium was still significantly abnormal on discharge in these patients. These sodium trends are summarized in Figure 2.

### 3.3. Intravenous Fluids

We examined the profile of intravenous fluid administration to see if there were differences which may explain the progressive water imbalance in some patients. Prior to the onset of moderate hypernatremia, 88% (159/180) of patients were receiving intravenous fluids (mean volume, 5.0 ± 4.2 L), and normal saline was the predominant fluid type given. After the onset of moderate hypernatremia, 83% (149/180) remained on intravenous fluids (mean volume, 3.0 ± 2.9 L). There was strong evidence that the difference in volume of prescribed fluids was significant (paired t-test, t_179_ = 5.9, *P* < 0.001). Even though there was an increasing percentage of low sodium and sodium free solutions used, over one-third of intravenous fluids prescribed remained as normal saline. The breakdown of the intravenous fluid composition is summarized in Table 3. A comparison of intravenous fluids by hypernatremia progression status is shown in Table 4.

### 3.4. Factors Associated with Severe Hypernatremia by Logistic Regression

From the univariable logistic regression analyses (Supplementary Materials Table S1), there were four variables of interest which were associated with severe hypernatremia: age (per 5 years, odds ratio [OR] 1.30, 95% confidence interval [CI]: 1.00–1.69, *P* = 0.053), volume of free water (OR 1.37, 95% CI: 1.10–1.72, *P* = 0.006), increase in serum creatinine (per 10 µmol/L, OR 1.30, 95% CI: 1.10–1.53, *P* = 0.002), and CKD stage (OR 2.52, 95% CI: 1.38–4.60, *P* = 0.003). The ordinal nature of the CKD relationship with hypernatremia suggested that parameterization as a continuous variable is preferred (test for linear trend, χ^2^(1) = 3.20, *P* = 0.001).

In the multivariable model, age was not statistically significant after adjusting for the other covariates (per 5 years, OR 1.23, 95% CI: 0.92–1.65, *P* = 0.16). Thus, in the final multivariable model, progression to severe hypernatremia was independently associated with CKD stage (OR 2.38, 95% CI: 1.26–4.50, *P* = 0.008), rise in serum creatinine (OR 1.29, 95% CI: 1.07–1.57, *P* = 0.009), and volume of free water prescribed (OR 1.29, 95% CI: 1.01–1.66, *P* = 0.047). The model showed good discrimination (c-statistic, 0.83) and was a good fit for the data (Hosmer–Lemeshow, χ^2^(8) = 8.1, *P* = 0.42).

On average, a one-unit increase in CKD stage was associated with a 2.38 times higher odds of progression to severe hypernatremia after the onset of moderate hypernatremia, after adjusting for the other covariates. On average, a 10 µmol/L increase in serum creatinine after the onset of moderate hypernatremia was associated with a 29% higher odds of progression to severe hypernatremia, after adjusting for the other covariates. Every liter of free water prescribed after the onset of moderate hypernatremia was associated with a 29% higher odds of progression to severe hypernatremia, on average, after adjusting for the other covariates.

### 3.5. Inpatient Mortality

Inpatient mortality was defined as a failure to survive to discharge or transfer. Overall, 52/180 (28.9%) of the included patients died during admission. Patients who progressed to develop severe hypernatremia were more likely to die during admission than those with moderate hypernatremia who did not progress (unadjusted OR 4.12, 95% CI: 1.47–11.5, *P* = 0.007).

## 4. Discussion

In this cohort study of hospital-acquired hypernatremia, we found that approximately 10% of patients who developed moderate hypernatremia (sodium 150 mmol/L) in hospital eventually progressed to severe hypernatremia (sodium 160 mmol/L or higher), and progression was associated with an increase odds of mortality. The main factors associated with the progression to severe hypernatremia were the CKD stage and a rise in serum creatinine. Patient age was less important in the multivariable model after accounting for CKD status. A greater volume of free water prescription was associated with progression to severe hypernatremia.

We have showed an association between CKD stage and progression of hospital-acquired hypernatremia, even after allowing for changes in serum creatinine. Our findings are supported by a previous study of hospital-acquired hypernatremia, where hypernatremia was more common in patients with lower eGFR [6]. Given the significant role of the kidney in sodium and water homeostasis, there is biological plausibility for this observation. Hyponatremia is more common than hypernatremia in the earlier stages of CKD, but this association is reversed with CKD progression as water handling becomes progressively impaired [17]. Patients with an eGFR below 30 mL/min/1.73m^2^ appear to have the highest risk and greater implications for mortality [17,18]. A large, longitudinal observational study of patients with non-dialysis CKD noted that the prevalence of hyponatremia was not correlated with CKD stage, but the prevalence of hypernatremia increased with advancing CKD [12]. Our data support this observation. Compared to patients without CKD, our Stage 3 CKD patients had an almost 4-fold higher odds of severe hypernatremia, while the odds were increased over 12-fold in our Stage 4 and 5 CKD patients. Thus, CKD stage may be a risk factor for both chronic, community-acquired hypernatremia as well as acute, hospital-acquired hypernatremia.

We found that a rise in serum creatinine was closely associated with progression to severe hypernatremia. We noted that the volume of fluid replacement was smaller after the onset of moderate hypernatremia. The type of intravenous fluids prescribed is also concerning, with a high percentage of normal saline used, leading to the onset of moderate hypernatremia, and almost 40% of the volume of intravenous fluids, on average, consisted of normal saline, even after serum sodium had reached 150 mmol/L. Palevsky et al. reported similar findings and suggested that hospital-acquired hypernatremia was an iatrogenic concern. In their patients who received intravenous fluids after hypernatremia onset (72%), nearly 40% received normal saline exclusively as well. Our data were consistent with this previous finding, suggesting that the volume and composition of fluids given was inadequate to prevent the further rise in serum sodium concentration. However, we also recognize that there are many causes of a rise in serum creatinine, which may be unaccounted for. For example, the use of nephrotoxic medications, sepsis, and heart failure are possible confounders commonly seen in hospitalized general medicine patients.

The reason for favoring and persisting with normal saline despite moderate hypernatremia could not be determined in our study. We can only speculate several possible reasons. There may be a delay in the recognition of hypernatremia, given it was not present on admission. The average length of stay in our patients was over two weeks and the initial onset of moderate hypernatremia may have been insidious, averaging one week after admission. Palevsky et al. also noted that patients with community-acquired hypernatremia received more appropriate fluid therapy than patients with hospital-acquired hypernatremia [5]. It is also possible that a fear of overly rapid correction of hypernatremia may explain why normal saline comprised 40% of the volume of fluids in patients with moderate hypernatremia in our study. The rate of correction is still debated but could have been quicker when there was an acute onset [19].

Although we found an association between a higher estimated free water volume of intravenous fluids and severe hypernatremia, this is not evidence of a cause–effect relationship. Rather, it may indicate that the higher volume of free water administered was a response to worsening hypernatremia. The difficulty with our analysis is that fluid treatment is dynamic and ongoing, rather than being a single intervention at one point in time (a time-dependent variable). However, we have left it in the multivariable logistic model as a covariate as it improved the precision of the model. Ultimately, we believe that the amount of free water prescribed was inadequate in the context of ongoing use of sodium-rich solutions and lower total volumes during progression of hypernatremia.

Our finding of a 29% hospital mortality rate in patients with hospital-acquired moderate to severe hypernatremia was lower than the 40 to 60% reported in other studies. One possibility is the relatively small proportion of patients with severe hypernatremia in our study (10%). It may also relate to the definitions used. We only examined in-hospital mortality, whereas 30-day mortality may be different. We found weak evidence of an association between age and progression to severe hypernatremia, but age did not seem to be predictive in the multivariable model. Given that CKD stage is correlated with age, it is likely that the effects of age have been accounted for by CKD. Alternatively, age may be a stronger risk factor for community-acquired or chronic hypernatremia than it is for severe hospital-acquired hypernatremia.

In this study, a history of dementia was not associated with progression to severe hypernatremia in hospital, even though dementia is a known risk factor for community-acquired hypernatremia. In our study, we excluded cases of community-acquired hypernatremia, which may have then resulted in an under-representation of patients with severe dementia in this cohort. In some studies, around 50% of patients who present to hospital with community-acquired hypernatremia had dementia [9,20]. One study noted a statistically significant ordinal relationship between the severity of hypernatremia and the proportion of patients with dementia in hospitalized patients, with a mean age of 81 years: mild hypernatremia (76%), moderate hypernatremia (83%), and severe hypernatremia (98%) [21]. Thus, it was possible that patient selection may have biased both the mortality rate and tests for the association of dementia with hypernatremia progression in our study. In our experience, dementia is not the only reason for hospitalized patients to not drink adequately. Given the elderly cohort, acute delirium can play a role, in addition to the loss of appetite and nausea from illness or medication side-effects. There are also reasons for enhanced water loss, such as fever and vomiting. Thus, many general medicine patients rely on supplemental intravenous fluids during their recovery. Lastly, the heterogeneity of the strength of the association between dementia and hypernatremia could also be influenced by the severity of the dementia. Most studies have analyzed dementia as a binary factor without distinguishing the severity or stage of the condition. Further studies would be useful to refine our understanding of the association between dementia and hypernatremia.

The major limitations of this study were its observational design, which meant that causality could not be established. The number of patients experiencing the outcome of interest was also small, as progression to severe hypernatremia during inpatient care was uncommon. It is possible that we made a type 2 error when testing the association of some of the factors examined. As patients were identified by discharge coding, it is possible that some patients with hypernatremia were missed. We were not able to incorporate data on urine composition such as volume, osmolality, and sodium concentration, as they were inconsistently performed. The extent of missing urine data precluded the use of multiple imputation to account for this. These data could have provided evidence for the volume status of patients. In some cases, it was possible that clinicians were less aggressive in correcting hypernatremia due to treatment limitations placed on patient care. However, given patients continued to undergo blood tests, and as such, were being actively treated, this is perhaps less likely. This study only assessed patients in a general medicine unit and the results may only be generalized to other general medicine inpatients. Definitions of hypernatremia are known to vary between studies and can be a source of heterogeneity of results, and our study is no exception. In particular, the cut-off for severe hypernatremia can be anything from 152 to 160 mmol/L in adults, and as high as 170 mmol/L in the pediatric population [22,23]. This lack of consensus can affect how studies are interpreted or compared.

## 5. Conclusions

Patients with CKD should be closely monitored for the development of severe hospital-acquired hypernatremia, particularly in those with an eGFR less than 30 mL/min/1.73m^2^. An increase in serum creatinine is associated with progression to severe hypernatremia in patients who have already reached a serum sodium of 150 mmol/L. The intravenous fluid prescription should be reviewed regularly in these patients to ensure an adequate volume of fluid with an appropriate amount of free water is given. Less normal saline should be used for replacement and maintenance fluids to prevent progression of hospital-acquired hypernatremia. Given the mortality implications of progression of hypernatremia, further research would be useful to understand why fluid prescription practices remain suboptimal.

## Figures and Tables

**Table 1:** Patient characteristics by hypernatremia progression status (*N* = 180).

Characteristics	All *n* = 180	Non-Progressors *n* = 163	Progressors *n* = 17
***Demographics***			
Age, median (IQR), years	81 (69–86)	80 (67–86)	84 (75–87)
Male sex, n (%)	109 (60.6)	98 (60.1)	11 (64.7)
Residential care, n (%)	55 (30.6)	49 (30.1)	6 (35.3)
***Comorbidities***			
Diabetes mellitus, n (%)	46 (25.6)	41 (25.2)	5 (29.4)
Functional disability, n (%)	10 (5.6)	9 (5.5)	1 (5.9)
Dementia, n (%)	33 (18.3)	31 (19.0)	2 (11.8)
Intensive care encounter, n (%)	81 (45.0)	75 (46.0)	6 (35.3)
***Chronic kidney disease*** ^1^			
No/Stage 1 (eGFR>90), n (%)	40 (22.3)	40 (24.7)	0 (0)
Stage 2 (eGFR 60-89), n (%)	48 (26.7)	44 (27.0)	4 (23.5)
Stage 3 (eGFR 30-59), n (%)	59 (33.0)	54 (33.1)	5 (29.4)
Stage 4/5 (eGFR<30), n (%)	33 (18.3)	25 (15.3)	8 (47.1)
***Diuretic use***			
Duration, mean (SD), days	2.2 (2.4)	2.3 (2.5)	1.4 (1.9)
Any diuretic ^2^, n (%)	108 (60.0)	98 (60.1)	10 (58.8)
Intravenous diuretic, n (%)	85 (47.2)	75 (46.0)	10 (58.8)
Oral diuretic, n (%)	55 (30.6)	52 (31.9)	3 (17.7)

^1^ eGFR based on CKD-EPI equation, in ml/min/1.73 m^2^. ^2^ Oral or intravenous diuretic. Abbreviations: IQR, interquartile range; eGFR, estimated glomerular filtration rate; SD, standard deviation.

**Table 2:** Serum sodium and creatinine by hypernatremia progression status.

Biochemistry	All Patients *n* = 180	Non-Progressors *n* = 163	Progressors *n* = 17
***On admission***			
Na^+^, mean (SD), mmol/L	138 (5)	138 (5)	138 (5)
Creatinine, mean (SD), µmol/L	154 (115)	149 (115)	193 (113)
Creatinine, median (IQR), µmol/L	123 (85–170)	120 (80–168)	194 (112–235)
***At onset of Na^+^ 150 mmol/L***			
Na^+^, mean (SD), mmol/L	152 (3)	152 (2)	155 (3)
Creatinine, mean (SD), µmol/L	147 (105)	145 (106)	168 (84)
Creatinine, median (IQR), µmol/L	122 (86–171)	120 (85–170)	145 (117–206)
Time from admission, mean (SD), days	7.3 (6.7)	7.2 (6.6)	7.7 (7.6)
***At peak of Na^+^***			
Na^+^, mean (SD), mmol/L	155 (4)	154 (3)	162 (3)
Creatinine, mean (SD), µmol/L	149 (105)	144 (105)	190 (90)
Creatinine, median (IQR), µmol/L	123 (84–174)	115 (80–163)	176 (142–219)
Time from onset, mean (SD), days	1.5 (3.0)	1.1 (2.5)	4.8 (4.8)

**Table 3:** Intravenous fluids seven days pre- and post-onset of moderate hypernatremia.

Fluid Type ^1^	*n* (%)	Volume in Liters Mean ± SD	Percent Total Fluids Mean ± SD
***Prior to onset of Na^+^ 150 mmol/L***			
0.9% NaCl	156 (86)	4.1 ± 2.8	75.6 ± 27.9
Hartmann’s ^2^	65 (36)	2.3 ± 1.7	14.0 ± 22.6
5% glucose	67 (37)	1.4 ± 1.3	7.7 ± 14.1
4% glucose - 0.18% NaCl	2 (1)	2.0 ± 0.0	0.5 ± 4.7
4% albumin	20 (11)	1.5 ± 1.5	2.2 ± 7.4
***After onset of Na^+^ 150 mmol/L***			
0.9% NaCl	95 (52)	2.0 ± 1.6	38.4 ± 36.0
Hartmann’s	26 (14)	1.7 ± 1.1	7.5 ± 20.3
5% glucose	120 (66)	2.4 ± 1.8	53.5 ± 39.6
4% glucose - 0.18% NaCl	8 (4)	1.9 ± 1.1	2.8 ± 14.0
0.45% NaCl	1 (<1)	2.0 ± 0.0	0.3 ± 4.0
4% albumin	8 (4)	1.6 ± 1.4	1.1 ± 4.8

^1^ Categories of fluids are not mutually exclusive as patients may have received more than one type of intravenous fluids. ^2^ Equivalent to Ringer’s lactate solution. NaCl = Sodium chloride, Na^+^ = serum sodium, SD = standard deviation.

**Table 4:** Volume (liters) of free water by hypernatremia progression status.

Fluid Type	All Mean ± SD	Non-Progressors Mean ± SD	Progressors Mean ± SD
***Prior to onset of Na^+^ 150 mmol/L***			
Mostly free water ^1^	0.5 ± 1.0	0.5 ± 1.0	0.7 ± 1.7
Little free water ^2^	4.5 ± 3.8	4.4 ± 3.7	4.9 ± 4.3
Total fluids	5.0 ± 4.2	4.9 ± 4.2	5.6 ± 4.7
***After onset of Na^+^ 150 mmol/L***			
Mostly free water ^1^	1.7 ± 1.9	1.5 ± 1.8	2.9 ± 2.5
Little free water ^2^	1.4 ± 2.1	1.4 ± 2.1	1.1 ± 1.7
Total fluids	3.0 ± 2.9	2.9 ± 2.8	4.1 ± 3.6

^1^ Includes 5% glucose and 4% glucose-0.18% NaCl solutions. ^2^ Includes 0.9% NaCl, Hartmann’s (Ringer’s lactate), 0.45% NaCl, 4% albumin.

## Supplementary Materials

The following are available online at https://www.mdpi.com/1010-660X/56/7/358/s1, Table S1: Univariable logistic regression on severe hypernatremia.
