# Supplementary material for: Progression to Severe Hypernatremia in Hospitalized General Medicine Inpatients: An Observational Study of Hospital-Acquired Hypernatremia
Source: Medicina (Kaunas). 2020 Jul 17;56(7):358. doi: 10.3390/medicina56070358 (PMC7404557; doi:10.3390/medicina56070358)
Supplement: Supplementary file 1 [file medicina-56-00358-s001.pdf]

Table S1: Univariable logistic regression on progression to severe hypernatremia

Factor/variable	Odds ratio	95% CIs	*P* value
Age, per 5 years	1.30	1.00-1.69	0.053
Male sex	1.22	0.43-3.45	0.71
Residential care	1.27	0.44-3.62	0.66
Dementia	0.57	0.12-2.61	0.47
Disability	1.07	0.13-8.99	0.95
Diabetes mellitus	1.24	0.41-3.73	0.70
CKD stage as continuous variable	2.52	1.38-4.60	0.003
Stage 4/5 CKD	4.91	1.73-13.9	0.003
Vomiting/diarrhoea	0.81	0.17-3.79	0.79
Thickened fluids	1.05	0.32-3.41	0.94
Fasting duration, days	1.01	1.00-1.03	0.11
Sepsis	1.21	0.44-3.30	0.71
Overall diuretic use	0.95	0.34-2.62	0.92
Intravenous diuretic use	1.68	0.61-4.62	0.32
Admission Cr, per 10 µmol/L	1.03	0.99-1.06	0.14
Intensive care encounter	0.64	0.23-1.81	0.40
Onset Cr, per 10 µmol/L	1.02	0.98-1.06	0.39
Peak Cr, per 10 µmol/L	1.03	0.99-1.07	0.11
Pre-onset change in Cr, per 10 µmol/L	0.98	0.93-1.03	0.35
Pre-onset no IV fluids	1.01	0.21-4.77	0.99
Pre-onset total IV fluid volume, Litres	1.04	0.93-1.16	0.52
Pre-onset *free water*[1] volume, Litres	1.20	0.83-1.76	0.34
Post-onset no IV fluids	1.03	0.28-3.84	0.96
Post-onset total IV fluid volume, Litres	1.12	0.97-1.29	0.14
Post-onset *free water*[1] volume, Litres	1.37	1.10-1.72	0.006
Post-onset change in Cr, per 10 µmol/L	1.30	1.10-1.53	0.002

[1] For estimation purposes, includes 5% glucose and 4% glucose/0.18% NaCl solutions.

Abbreviations: Cr, Creatinine; CKD, Chronic kidney disease.
